# Evolutionary relationships within the lamioid tribe Synandreae (Lamiaceae) based on multiple low-copy nuclear loci

**DOI:** 10.7717/peerj.2220

**Published:** 2016-07-20

**Authors:** Tilottama Roy, Nathan S. Catlin, Drake M.G. Garner, Philip D. Cantino, Anne-Cathrine Scheen, Charlotte Lindqvist

**Affiliations:** 1 Department of Biological Sciences, University at Buffalo, Buffalo, NY, United States; 2 Department of Environmental and Plant Biology, Ohio University, Athens, OH, United States; 3 Museum of Archaeology, University of Stavanger, Stavanger, Norway; 4 Current affiliation: Department of Ecology and Evolutionary Biology, University of Michigan, Ann Arbor, MI, United States; 5 Current affiliation: Department of Biology, University of Florida, Gainesville, FL, United States

**Keywords:** Synandreae, Biogeography, Phylogeny, Stachydeae, Nuclear markers, North America, *Physostegia*, Lamiaceae

## Abstract

The subfamily Lamioideae (Lamiaceae) comprises ten tribes, of which only Stachydeae and Synandreae include New World members. Previous studies have investigated the phylogenetic relationships among the members of Synandreae based on plastid and nuclear ribosomal DNA loci. In an effort to re-examine the phylogenetic relationships within Synandreae, the current study incorporates data from four low-copy nuclear loci, *PHOT1*, *PHOT2*, *COR*, and *PPR*. Our results confirm previous studies based on chloroplast and nuclear ribosomal markers in supporting the monophyly of tribe Synandreae, as well as sister relationships between *Brazoria* and *Warnockia*, and between that pair of genera and a monophyletic *Physostegia*. However, we observe incongruence in the relationships of *Macbridea* and *Synandra*. The placement of Synandreae within Lamioideae is poorly resolved and incongruent among different analyses, and the sister group of Synandreae remains enigmatic. Comparison of the colonization and migration patterns corroborates a single colonization of the New World by Synandreae during the Late Miocene/Tortonian age. This is in contrast to the only other lamioid tribe that includes New World members, Stachydeae, which colonized the New World at least twice—during the mid-Miocene and Pliocene. Edaphic conditions and intolerance of soil acidity may be factors that restricted the distribution of most genera of Synandreae to southeastern and south–central North America, whereas polyploidy could have increased the colonizing capability of the more wide-ranging genus, *Physostegia*.

## Introduction

**Figure 1:**
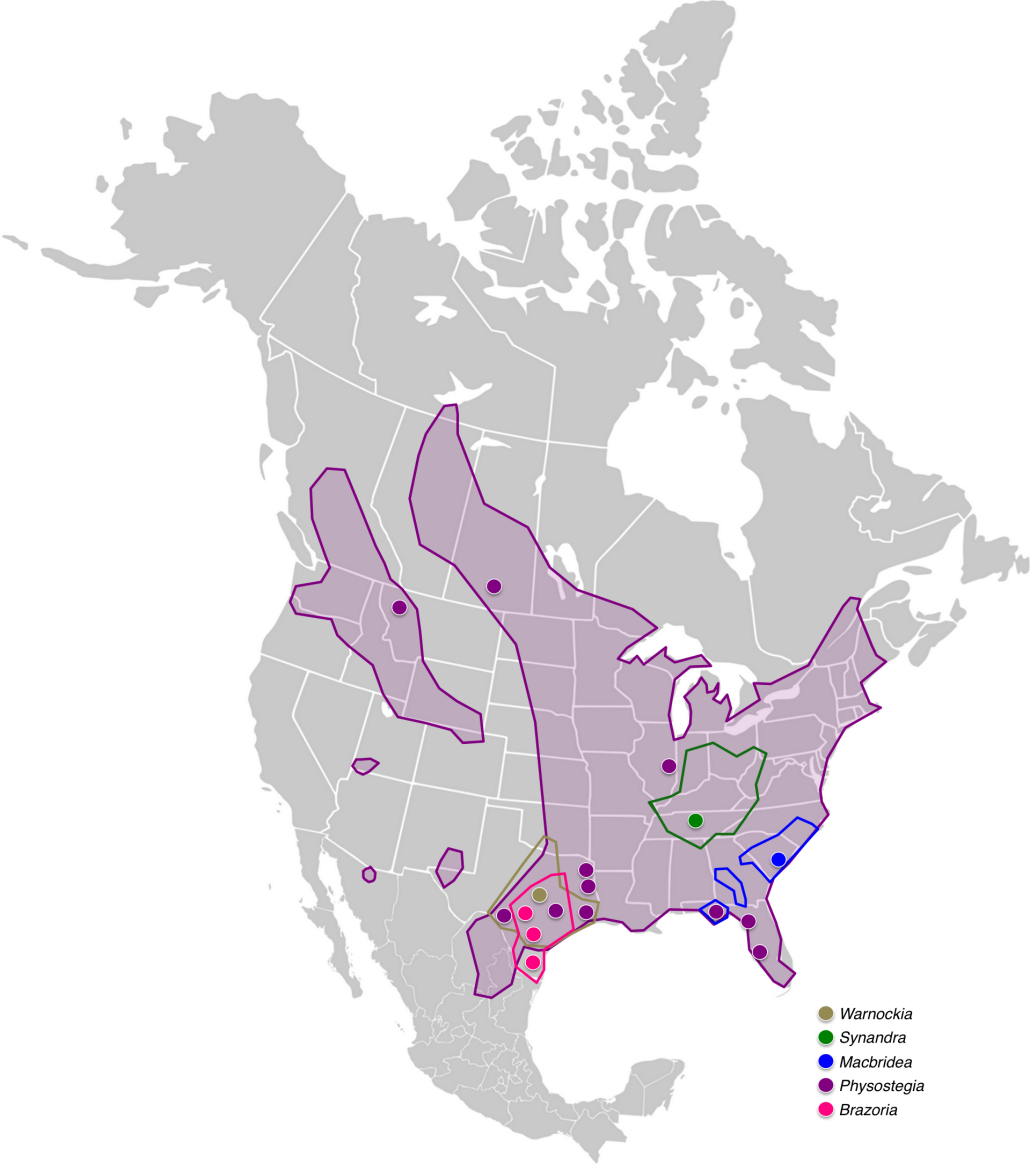
Distribution map of Synandreae in North America. Geographical ranges of the Synandreae genera are outlined and the geographic locations of the accessions included in this study are indicated with circles. See key for color coding of the genera. Distributions were based on Cantino (1980), Cantino (1982) and Turner (1996), and BONAP’s North American Plant Atlas (NAPA; http://www.bonap.org).

The angiosperm family Lamiaceae has a worldwide distribution, comprising ∼7,200 species in approximately 240 genera (Bentham, 1876; Harley et al., 2004). Lamiaceae is subdivided into seven subfamilies, of which Lamioideae, the second largest, exhibits an impressive ecological and taxonomic diversity (Scheen et al., 2010; Bendiksby et al., 2011; Roy & Lindqvist, 2015). Most members of Lamioideae have been classified into ten tribes, with the majority of the species inhabiting Eurasia and Africa. Approximately 113 species, however, are native to the New World, and they are members of just two tribes: Stachydeae and Synandreae (Scheen et al., 2010; Roy et al., 2013; Roy et al., 2015). Considerable molecular phylogenetic work has recently been performed in Stachydeae (Lindqvist & Albert, 2002; Salmaki et al., 2013; Roy et al., 2013; Roy et al., 2015), and it has been suggested that the New World members of the genus *Stachys* colonized the Americas twice, first during the mid-Miocene and later during the early Pliocene (Roy et al., 2013; Roy et al., 2015). The focus of the current study is Synandreae, the other lamioid tribe represented in the New World (Fig. 1), comprising five genera: *Synandra* Nutt., *Macbridea* Elliott ex Nutt., *Brazoria* Engelm & A. Gray, *Warnockia* MW Turner, and *Physostegia* Benth.

All five genera of Synandreae are herbs with relatively large flowers (for Lamiaceae), which are sessile or short-pedicellate in racemoid inflorescences. Corolla color ranges from white (*Macbridea alba*, *Synandra*, and some *Physostegia* species) to lavender (*Macbridea caroliniana*, *Brazoria*, *Warnockia*, and most *Physostegia* species). The anther thecae are either narrow apically to a sharp point (*Synandra*) or bear one or more teeth along the suture. Monotypic *Synandra hispidula* (2*n* = 18) is a biennial of mesic woodlands in the eastern United States, mostly occurring in the Appalachian region (Fig. 1). It differs from the rest of the tribe in having long-petiolate, cordate-ovate leaves. *Macbridea* (2*n* = 18) comprises two species of rhizomatous perennial herbs of wetlands and pine savannas in the southeastern United States (Fig. 1). *Macbridea* flowers are tightly packed into terminal and sub-terminal capitate glomerules, unlike the elongate inflorescences of the other four genera, and its three-lobed calyx is distinctive. *Brazoria* (2*n* = 28) comprises three species of annuals of sandy soils in eastern and central Texas (Fig. 1), with an erect and deeply bifid upper corolla lip (Turner, 1996). Monotypic *Warnockia scutellarioides* (2*n* = 20) is an annual of calcareous soils in Texas, southern Oklahoma, and northern Mexico (Coahuila) (Turner, 1996) (Fig. 1). *Physostegia* (2*n* = 38 and 76), with 12 species of perennials, is the most widespread genus of Synandreae, ranging from Northern Canada to Northern Mexico (Fig. 1) and growing in diverse habitats and a wide range of soil conditions (Cantino, 1982). *Physostegia virginiana* is often grown as an ornamental and has become naturalized in some areas. *Physostegia* is the only genus of Synandreae with an actinomorphic, five-lobed calyx.

Bentham (1848) described subtribe Melittidinae (“Melittieae”), comprising the monotypic European genus *Melittis* and the North American genera *Brazoria*, *Synandra*, *Macbridea*, and *Physostegia*. Bentham (1876) and Briquet (1895–1897) added the Asian genus *Chelonopsis* to this subtribe but transferred *Brazoria* to Scutellariinae and Prunellinae, respectively. Cantino (1985a) and Abu-Asab & Cantino (1987) considered Melittidinae to include *Brazoria*, and Turner (1996) segregated *Warnockia* from *Brazoria*. However, morphological and karyological studies (Cantino, 1982; Cantino, 1985a) and investigation of leaf anatomy (Abu-Asab & Cantino, 1987), palynology (Abu-Asab & Cantino, 1994), and pericarp structures (Ryding, 1994) were unable to provide synapomorphies supporting the monophyly of Melittidinae. Furthermore, molecular phylogenetic studies demonstrated the non-monophyly of Melittidinae (Scheen et al., 2008; Scheen et al., 2010; Bendiksby et al., 2011; Salmaki et al., 2013; Roy & Lindqvist, 2015). Scheen et al. (2008) found that, rather than grouping with the North American endemics, *Melittis melissophyllum* grouped with *Stachys*, and *Chelonopsis* grouped with the Asian genus *Gomphostemma*. These studies also demonstrated the monophyly of a group comprising the North American endemics (*Brazoria*, *Warnockia*, *Synandra*, *Macbridea*, and *Physostegia*). Since *Melittis* is not part of this clade, it could no longer be named Melittidinae and was instead named tribe Synandreae (Scheen et al., 2008). Since the study by Scheen et al. (2008) was based on chloroplast and nuclear ribosomal DNA markers, the goal of the current study is to investigate the phylogenetic relationships among the members of Synandreae based on low-copy nuclear markers.

With the availability of improved technologies and universal primers, there has been a shift from plastid and ribosomal loci towards the use of low-copy nuclear genes (Mort & Crawford, 2004) in investigations of interspecific phylogenetic relationships because they often have a higher rate of evolution, leading to higher resolution in species-level phylogenies. Furthermore, maternally inherited plastid DNA, as a single linkage group, can only provide the genealogical history of one parent and thus cannot provide any information on hybrid species histories. Although nuclear ribosomal DNA (e.g., ITS, ETS, and 5S-NTS) is biparentally inherited, these data do not always provide reliable markers for the reconstruction of hybrid speciation and resolution of phylogenetic histories due to concerted evolution and homogenization (Wendel, Schnabel & Seelanan, 1995). Hence, the true evolutionary relationships among closely related taxa may be confounded. Also, in situations where speciation has taken place rapidly, as may be the case within Synandreae, genomic DNA may not have undergone enough divergence to resolve a phylogeny with only one locus (Seehausen, 2004). In such cases, multiple independent nuclear loci may provide the variability necessary to make a more accurate estimation of phylogenetic relationships (Sang, 2002; Hughes, Eastwood & Bailey, 2006). However, low-copy nuclear genes are not devoid of shortcomings. Some of the issues encountered while dealing with low-copy nuclear loci include presence of paralogous copies, incomplete lineage sorting, and gene tree/species tree incongruence due to hybridization and introgression. Hence, these factors should always be taken into account when drawing conclusions on evolutionary relationships.

In this study, we have analyzed data from four low-copy nuclear loci: two *PHOT* gene duplicates (*PHOT1* and *PHOT2*), *COR* (cold acclimation protein), and the *PPR* (pentatricopeptide repeat) region AT3G09060. The *PHOT* genes are responsible for encoding the blue and ultra-violet-A light receptor of plants involved in the process of phototropism (Christie et al., 1998), chloroplast relocation (Jarillo et al., 2001; Kagawa et al., 2001), and the regulation of stomatal openings (Kinoshita et al., 2001). Two *PHOT* loci are present in most angiosperms (*PHOT1* and *PHOT2*), resulting from a duplication event predating the divergence between monocots and tricolpates (Briggs et al., 2001). The two *PHOT* gene duplicates have accumulated a sufficiently large number of nucleotide substitutions since their divergence to be distinct from each other, which is important for overcoming orthology/paralogy issues when being utilized in phylogenetic analyses (Fitch, 1970). The two paralogs have been shown to be so variable that their intron regions are unalignable with each other and hence can be treated as two separate markers. Due to the presence of many small, relatively conserved exon regions, separated by variable introns, it has been suggested that the amount of information that can be collected from these loci is high relative to the effort that is applied to work with them (Yuan & Olmstead, 2008). Also, through the investigation of these two paralogs, the mode of intron evolution can be observed across closely related species, such as members of Synandreae. All these factors make the *PHOT* gene duplicates ideal for use in our current study. The *COR* locus also consists of intron regions flanked by exons that provide conserved primer binding sites (EPIC markers; Curto et al., 2012; Thomson et al., 2008). Curto and colleagues (2012) have shown from their study of *Micromeria* (Lamiaceae) that this locus can be phylogenetically informative, providing a substantial amount of variation among closely related species. Lastly, the *PPR* gene family encodes a group of proteins with short helical repeats that are arranged in stacks, forming extended surfaces (Geddy & Brown, 2007; Barkan & Small, 2014). Previous studies (Yuan et al., 2009; Yuan et al., 2010; Crowl et al., 2014) and our own study on Lamioideae (Roy & Lindqvist, 2015) have demonstrated the *PPR* loci as a useful marker to reconstruct phylogenetic relationships involving rapidly radiating taxa. In addition to the low-copy nuclear markers, we also incorporated chloroplast DNA (cpDNA) data from previous studies (Scheen et al., 2008; Scheen et al., 2010; Bendiksby et al., 2011) for four regions (*mat*K, *rp*s16, *trn*L intron, and *trn*L-F spacer) to generate a more comprehensive multispecies coalescent tree.

The goals of this study included (1) assessing the monophyly of tribe Synandreae, (2) further clarifying relationships within Synandreae, (3) investigating the historical biogeography of Synandreae, including its introduction into the New World, and (4) comparing the migration and diversification patterns of Synandreae with those of tribe Stachydeae, the only other lamioid tribe with endemic New World species.

## Methods

### Taxon sampling, DNA extraction, amplification, and sequencing

Leaf material was collected from specimens held at the following herbaria: BISH, C, GH, LL, O, TEX, UNA, UPS, and US (herbarium acronyms follow Holmgren, Holmgren & Barrett, 1990). All taxon names in this study follow the “World checklist of Lamiaceae and Verbenaceae” (Govaerts et al., 2013). DNA was extracted from silica-dried leaves or from herbarium specimen leaf fragments using the DNeasy Plant Mini Kit (Qiagen, Hilden, Germany) following the manufacturer’s instructions. DNA sequence data were collected for accessions from all five genera, representing 17 of the 19 recognized species of Synandreae (excluding *Macbridea alba* and *Physostegia intermedia*) (Table 1). Furthermore, 22 additional lamioid outgroup species were selected based on previous studies (Scheen et al., 2010; Bendiksby et al., 2011). *Scutellaria hirta* was included to root the trees since many studies have shown Scutellarioideae and Lamioideae to be closely related (Wagstaff et al., 1998; Scheen et al., 2010; Bendiksby et al., 2011; Li et al., 2012; Chen et al., 2014).

**Table 1:** List of taxa and voucher information. Herbaria abbreviations follow the Index Herbariorum (Holmgren, Holmgren & Barrett, 1990). One individual per species was included. For *PHOT1*, *PHOT2*, *COR*, and *PPR*, the number of sequences indicate the number of clones per species. cpDNA regions included: a, *matK*; b, *rps16*; c, *trnL*; d, *trnL-F*. For the cpDNA sequences the same accession of each of the species listed was sequenced.

Taxon names	Tribe/subfamily	Voucher information	Geographic distribution	GenBank accession numbers				
				*PHOT*1	*PHOT*2	*COR*	*PPR*	cpDNA
**Ingroup**								
*Brazoria arenaria* Lundell	Synandreae	M.W. Turner 25 (TEX)	USA	KT716942	N/A	N/A	KT378319 KT378320 KT378321	N/A
*Brazoria enquistii* M.W.Turner	Synandreae	M.W. Turner 61 (TEX)	Texas, USA	KT716996 KT716997	KT717006 KT717007	KT716873 KT716874	KT378322	a.HQ911432 b.HQ911600 c.EF546966 d.EF546889
*Brazoria truncata* (Benth.) Engelm. & A.Gray	Synandreae	D.S. Corell 1605 (GH)	Texas, USA	KT716988 KT716989 KT716990 KT716991	N/A	N/A	N/A	N/A
*Macbridea caroliniana* (Walter) S.F.Blake	Synandreae	R.K. Godfrey & R.M. Tryon 741 (GH)	USA	KT716986 KT716987	KT717004 KT717005	KT716881 KT716882	KT378355 KT378356	c.EF546963 d.EF546887
*Physostegia angustifolia* Fernald	Synandreae	C.L. Lundell & A.A. Lundell 16031 (US)	Texas, USA	KT716971 KT716973 KT716974	KT717023 KT717024	KT716896	KT378384 KT378385	a.HQ911434 c.EF546942 d.EF546865
*Physostegia correllii* (Lundell) Shinners	Synandreae	D.S. Corell & I.M. Johnston 19427 (LL)	Texas, USA	KT716955 KT716956 KT716957	KT717022	KT716897 KT716898	N/A	N/A
*Physostegia digitalis* Small	Synandreae	P.D. Cantino 1076 (GH)	Texas, USA	KT716974	KT717012 KT717013	KT716899 KT716900 KT716901 KT716902	KT378386 KT378387	c.EF546945 d.EF546945
*Physostegia godfreyi* P.D.Cantino	Synandreae	R.K. Godfrey 77073 (GH)	Florida, USA	KT716958 KT716959 KT716960	KT717016 KT717017	KT716903	N/A	N/A
*Physostegia ledinghamii* (Boivin) P.D.Cantino	Synandreae	V.L. Harms 34491 (GH)	Saskatchewan, Canada	KT716975 KT716976	KT717010 KT717011	KT716904 KT716905 KT716906 KT716907	KT378388 KT378389	a.HQ911435 c.EF546950 d.EF546874
*Physostegia leptophylla* Small	Synandreae	P.D. Cantino 1026 (GH)	Florida, USA	KT716961 KT716962	KT717018 KT717019	KT716908	KT378390 KT378391	c.EF546952 d.EF546875
*Physostegia longisepala* P.D.Cantino	Synandreae	L.E. Brown 13523 (TEX)	Texas, USA	KT716963 KT716964	KT717027 KT717028	N/A	N/A	N/A
*Physostegia parviflora* Nutt. ex A.Gray	Synandreae	M. Mooar 13667 (GH)	Montana, USA	KT716965 KT716966	KT717020 KT717021	KT716892 KT716893 KT716894 KT716895	KT378392	c.EF546954 d.EF546877
*Physostegia pulchella* Lundell	Synandreae	Wm.F. Mahler 8530 (GH)	Texas, USA	KT716967 KT716968	KT717025 KT717026	KT716909	KT378393	a.HQ911440 c.EF546956 d.EF546879
*Physostegia purpurea* (Walter) S.F.Blake	Synandreae	P.D. Cantino 1007 (GH)	USA, Florida, Sarasota Co.	KT716969 KT716970	N/A	KT716910 KT716911 KT716912 KT716913 KT716914 KT716915	N/A	N/A
*Physostegia virginiana* (Walter) S.F.Blake	Synandreae	P.D. Cantino 1007 (GH)	Florida, USA	KT716977 KT716978	KT717029 KT717030	KT716916 KT716917 KT716918	KT378394 KT378395	a.HQ911437 c.HQ911671 d.EF546884
*Synandra hispidula* (Michx.) Baill.	Synandreae	V.E. McNeilus 97–143 (GH)	Tennessee, USA	KT716998 KT716999	KT717002 KT717003	KT716924 KT716925 KT716926 KT716927	KT378434 KT378435	a.HQ911427 b.HQ911597 c.EF546970 d.HQ911737
*Warnockia scutellarioides* (Engelm. & A.Gray) M.W.Turner	Synandreae	M.W. Turner 67 (TEX)	Texas, USA	KT717000 KT717001	KT717008 KT717009	KT716928 KT716929 KT716930	KT378436 KT378437	a.HQ911429 b.HQ911599 c.EF546971 d.EF546895
**Outgroup**								
*Achyrospermum carvalhoi* Gürke	Pogostemoneae	E. Farkas & T. Pocs 86604 (UPS)	Tanzania	KT716933 KT716934	N/A	N/A	KT378299 KT378300	N/A
*Acrotome inflata* Benth.	Leucadeae	G.L. Maggs & L. Guarino 1072 (UPS)	Namibia	N/A	N/A	KT716867 KT716868 KT716869	KT378302 KT378303 KT378304	N/A
*Acrotome pallescens* Benth.	Leucadeae	I. Ortendahl 105 (UPS)	Namibia	N/A	KT717048	KT716870 KT716871 KT716872	N/A	N/A
*Ballota nigra* L. subsp. *ruderalis* (Sw.) Briq.	Marrubieae	M. Bendiksby & A.-C. Scheen (O)	Greece	KT716937 KT716938	KT717039 KT717040 KT717041	N/A	KT378308 KT378309 KT378310	N/A
*Ballota pseudodictamnus* (L.) Benth.	Marrubieae	M. Bendiksby & A.-C. Scheen 0420 (O)	Greece	KT716939 KT716940	KT717042 KT717043 KT717044	N/A	KT378311 KT378312 KT378313	N/A
*Betonica macrantha* K.Koch	N/A	D. McNeal et al. 161 (C)	Georgia	KT716941	KT717049 KT717050	N/A	KT378314 KT378315	N/A
*Galeopsis pyrenaica* Bartl.	N/A	P. Montserrat & al. 141487 (C)	Spain	KT716943 KT716944 KT716945 KT716946	N/A	N/A	KT378335	N/A
*Gomphostemma javanicum* (Blume) Benth.	Gomphostemmateae	R.G. Olmstead 93-38	S. China to SE Asia	KT716935 KT716936	KT717045 KT717046 KT717047	KT716875 KT716876	KT378336 KT378337	N/A
*Leonotis nepetifolia* (L.) R.Br.	Leucadeae	R. Abdallah et al. 493 (UPS)	Tanzania	KT716947 KT716948	N/A	KT716877 KT716878	KT378342	N/A
*Leucas inflata* Benth.	Leucadeae	V. Goloskokov s.n., 15 May 1963 (C)	Ethiopia	KT716949 KT716950	N/A	KT716879 KT716880	KT378345 KT378346 KT378347	N/A
*Marrubium peregrinum* L.	Marrubieae	A. Strid 33875 (C)	Greece	KT716942	N/A	KT716883 KT716884 KT716885	KT378357 KT378358	N/A
*Phlomis fruticosa* L.	Phlomideae	C. Mathiesen & J.M. Taylor 81 (National Collection of *Phlomis*, UK)	Sardegna (Italy) to Transcaucasus	KT716951 KT716952	KT717051 KT717052	KT716886 KT716887 KT716888	KT378372 KT378373 KT378374	N/A
*Phlomis tuberosa* L.	Phlomideae	C. Mathiesen & J.M. Taylor 88 (National Collection of *Phlomis*, UK)	EC Europe to China and Mongolia	KT716953 KT716954	N/A	KT716889 KT716890 KT716891	KT378377 KT378378 KT378379	N/A
*Phyllostegia kaalaensis* St.John	Stachydeae	S. Perlman 6117 (BISH)	Hawaii/O’ahu	KT716980 KT716981	KT717033 KT717034	N/A	N/A	N/A
*Scutellaria hirta* Sm.	Scutellarioideae	M. Bendiksby & A.-C. Scheen 0411 (O)	Greece	KT716931 KT716932	N/A	KT716919 KT716920 KT716921	KT378403 KT378404	N/A
*Stachys chamissonis* Benth.	Stachydeae	C. Lindqvist 10-02 (UB)	W. Canada to W. USA	KT716982 KT716983	KT717035 KT717036	N/A	N/A	N/A
*Stachys sylvatica* L.	Stachydeae	C. Lindqvist & V.A. Albert 358 (UNA)	Macaronesia, Europe to W Himalaya (cultivar)	N/A	N/A	KT716922	KT378410 KT378411	N/A
*Stachys bullata* Benth.	Stachydeae	C. Lindqvist 11-02 (UB)	W.California/California, Monterey Co.	KT716979	KT717037 KT717038	N/A	KT378415	N/A
*Stenogyne kamehamehae* Wawra.	Stachydeae	S. Perlman 6933 (BISH)	Hawaii	KT716984 KT716985	KT717031 KT717032	N/A	KT378430 KT378431	N/A
*Stenogyne calaminthoides* A.Gray	Stachydeae	C. Lindqvist & V. A. Albert 82 (NY)	Hawaii	N/A	N/A	KT716923	N/A	N/A

**Notes.**

N/A Not available/included

For amplification of the two *PHOT* loci, we used primers previously published by Yuan & Olmstead (2008). For the *PHOT1* locus, we utilized the primers 10F (‘5′-ATTGGAGTSCAAYTAGATGGAAG-‘3′) and 12R (‘5′-TCCACAAGTCCTCTGGTTTCT-‘3′). For the *PHOT2* locus, due to difficulty in amplification of the entire locus, we amplified two separate fragments and treated them initially as two separate loci, labeling them *PHOT2A* and *PHOT2B*. For the amplification of *PHOT2A*, we utilized the primers 10F (‘5′- GATGGAAGTGATMATKTGGAAC-‘3′) and 12R (‘5′-AGCCCACAGGTCYTCTGGTCTC-‘3′), whereas *PHOT2B* was amplified with primers 12F (‘5′-GAGACCAGARGACCTGTGGGCT’-‘3′) and 14R (‘5′- GATTTRTCCATTG CTTTCATGGC-‘3′). The *COR* locus was amplified using the following primers previously published by Curto et al. (2012): forward primer (‘5′-CTCGAATGTGTTCCTGCAG-‘3′) and reverse primer (‘5′- CACATCCCTCTTAGTCCCATAC-‘3′). Amplification and sequencing of *PPR* is described in Roy & Lindqvist (2015). All loci were amplified separately using a GeneAmp PCR System 9700 (Applied Biosystems, Foster City, CA, USA) using a touchdown method with the following thermocycling profile: hold for 10 min at 95 °C; 10 cycles of 1 min at 95 °C, 1 min at 60 °C and decreasing the temperature by 1 °C every cycle, 1 min at 72 °C; followed by 35 cycles of 1 min at 94 °C, 1 min at 50 °C, 1 min at 72 °C; and a final extension of 1 min at 72 °C. In certain cases when this touchdown method failed to amplify our locus of interest, a modified touchdown method was used, where the annealing temperature started at 55 °C and decreased by 1 °C every cycle. PCR products were purified using the QIAquick PCR purification kit (Qiagen, Hilden, Germany) following the manufacturer’s instructions. All the PCR reactions were performed in 25 µl volumes with the AmpliTaq DNA Polymerase buffer II kit (Applied Biosystems, Foster City, CA, USA) using 8.5 µl of de-ionized water, 2.5 µl each of buffer, MgCl_2_, Bovine Serum Albumin (BSA), tetramethylammonium chloride (TMACL) and dimethyl sulfoxide (DMSO), 0.5 µl each of the primers, 0.2 µl AmpliTaq gold and 2 µl genomic DNA. When a single clear band was visible from gel electrophoresis, PCR products were purified using the QIAquick PCR purification kit (Qiagen, Hilden, Germany) following the manufacturer’s instructions. When multiple bands were present, gel extraction and purification was done using the QIAquick Gel Extraction Kit (Qiagen, Hilden, Germany).

All PCR products generated were further cloned using the Qiagen PCR cloning kit (Qiagen, Hilden, Germany) following the manufacturer’s instructions, with the exception that 25 µL competent cells were used to transform each ligation reaction. Transformed clones were incubated overnight at 37 °C. Up to 12 positive clones were picked per individual, with the average number of clones varying between 2 and 4 per locus. PCR reactions were prepared in 25 µL volumes with the AmpliTaq DNA Polymerase buffer II kit (Applied Biosystems, Foster City, CA, USA) using 2.5 µL buffer, 2.5 µL MgCl2, 1.0 µL dNTP, 0.6 µL each of M13F and M13R primers, and 0.2 µL of AmpliTaq polymerase. All PCR products were examined by gel electrophoresis on 1% agarose gels, and positive PCR amplified products were sequenced in one direction using SP6 or T7 primers at the University of Washington High Throughput Genomics Center, Seattle, USA.

### Phylogenetic tree reconstruction

All sequences generated were edited and assembled in the program Sequencher v.4.7 (Gene Codes, Ann Arbor, Michigan, USA) and aligned with ClustalX v.2 (Larkin et al., 2007) or MAFFT (EMBL-EBI); the alignments were manually adjusted in BioEdit (Hall, 1999). Gaps were treated as missing data, and indels were not coded. We evaluated evidence of recombination using the Phi test (Bruen, Philippe & Bryant, 2006) in Splitstree v.4.13.1 (Huson, 1998). Initial Bayesian and maximum likelihood (ML) analyses were performed on the two *PHOT*2 regions, *PHOT*2*A* and *PHOT*2*B* (see above), separately, but since their topologies were compatible, the datasets generated from these two regions were concatenated in the program WINCLADA (Nixon, 1999) before running further phylogenetic analyses. Phylogenetic relationships were examined for the three loci, *PHOT1*, *PHOT2* and *COR*, separately, using Bayesian inference conducted in either MrBayes v.3.1.2 or 3.2.2 (Huelsenbeck & Ronquist, 2001) using CIPRES XSEDE (Miller, Pfeiffer & Schwartz, 2010). We utilized the model jumping feature in MrBayes, and allowed the best fit models to be sampled according to their posterior probabilities through the command nst = mixed. We ran the Bayesian analysis with two Markov Chain Monte Carlo (MCMC) chains for 10 million generations each. Convergence and mixing were monitored using Tracer 1.5 (Drummond & Rambaut, 2007). A burn-in of 25% was implemented. We also conducted ML analyses using the RAxML Blackbox webserver (Stamatakis, Hoover & Rougemont, 2008), or through the RAxML HPC Blackbox in the CIPRES portal (Miller, Pfeiffer & Schwartz, 2010), with 100 replicates. We initially ran jModeltest v.1.1 (Posada, 2008) to find the best fit model for each of our dataset. The HKY+G model was retrieved for the *PHOT1* loci. The TPM1uf+G and TrN+G models were retreived for *PHOT2*, and *COR*, respectively, but since these two models are not implemented in some of our analyses (see *BEAST analysis below), we utilized the next model (HKY+G) proposed with the highest score. Since our results included G (gamma model of rate heterogeneity), we incorporated it for all our ML analyses. However, we did not include the proportion of invariant sites (I), as this was not shown in any of our jModeltest results. We rooted the *COR* and *PHOT1* trees with *Scutellaria hirta* (not shown in figures), however, due to lack of sequence data for *S. hirta* for the *PHOT2* loci, we used *Gomphostemma javanicum* to root the *PHOT2* tree. Phylogenetic analyses of *PPR* alone are described in Roy & Lindqvist (2015).

In addition to analyzing our individual datasets, we also concatenated data from all the low-copy loci in the program WINCLADA (Nixon, 1999). We concatenated the arbitrary haplotype numbers for each gene and conducted a Bayesian analysis using BEAST v.1.8.3 (Drummond & Rambaut, 2007) and ML analysis through the RAxML HPC Blackbox in the CIPRES portal (Miller, Pfeiffer & Schwartz, 2010) on this dataset (with the same settings used for the individual datasets). We utilized the program PartitionFinder to partition our dataset (Lanfear et al., 2012) for selecting the partitioning schemes and nucleotide substitution models for our different gene regions. For our ML analyses, we ran 100 replicates, and added the gamma (G) parameter, but excluded the number of invariant sites (I). All four loci fit the model trN+G. However, since this model is not implemented in BEAST, we instead utilized GTR+G, which has the same parameters. Rate variation was modeled among branches using uncorrelated lognormal relaxed clocks (Drummond & Rambaut, 2007), with a single model for all genes. A Yule speciation process was used for the tree prior, and posterior distributions of parameters, including the tree, were estimated using MCMC sampling. We performed two replicate MCMC runs, with the tree and parameter values sampled every 5,000 steps over a total of 50 million generations. A maximum clade credibility tree was obtained using Tree Annotator within the BEAST v.1.8.3 package with a burn-in of 5,000 trees. Acceptable sample sizes and convergence to the stationary distribution were checked using Tracer 1.5 (Drummond & Rambaut, 2007).

### Coalescence analysis and network analysis

We implemented a multispecies coalescence model within the BEAST v.1.8.0 software package (Drummond & Rambaut, 2007) to further explore phylogenetic signals within Synandreae. *BEAST applies Bayesian MCMC analysis of the sequence data, jointly exploring gene trees and species trees to estimate the species tree posterior distribution under the assumption of the coalescence model. For this purpose, we incorporated sequences for all the members of Synandreae from the low-copy nuclear loci *PPR* (Roy & Lindqvist, 2015), along with *COR*, *PHOT1* and *PHOT2*, as well as from a concatenated dataset comprising sequences from the cpDNA regions *mat*K, *rp*s16, *trn*L intron, and *trn*L-F spacer obtained from previously published studies (Scheen et al., 2008; Scheen et al., 2010; Bendiksby et al., 2011). Previously, Scheen and colleagues (2008) carried out combined analyses of nrDNA and cpDNA datasets. However, since nrDNA suffer from issues of biased-concerted evolution, which may confound results from the species tree reconstruction, we decided not to include nrDNA data in the coalescence analysis. Datasets were pruned, keeping only members of Synandreae and taxa common to all of the loci. The nuclear loci were treated as unlinked. A relaxed molecular clock model for all the loci and HKY+G models of nucleotide substitution were applied for the nuclear loci, and the GTR+G model for the cpDNA regions. These models were derived from our jModeltest (Posada, 2008) results (see above for details). The tree prior was set to exponential, and other priors were kept to default values. Analyses were done for 10 million generations sampling every 10,000 generations. A relative proportion of the posterior samples from each Markov chain were discarded as burn-in, and trees were summarized in TreeAnnotator v.1.8.0 (Drummond & Rambaut, 2007). The resulting trees were then visualized in FigTree v.1.4.0 (Rambaut, 2008).

We also implemented a phylogenetic network method to analyze signals of reticulate evolution and character conflicts in our datasets. The network was created with Neighbor-Net (Huson & Bryant, 2006) in SplitsTree v.4.13.1 (Huson, 1998) using uncorrected *p*-distances. For this purpose, we utilized *PHOT1* and *PPR*, the two nuclear loci that have the highest representation in the various lamioid tribes, generating a concatenated dataset in WINCLADA (Nixon, 1999). For distance calculations, we chose the most parameterized model available in SplitsTree v.4.13.1 with an HKY85 model, transitions: transversions weighted 2:1, gamma model of rate heterogeneity, and base frequencies estimated empirically.

### Ancestral area reconstruction and divergence timing of Synandreae

For our ancestral area reconstruction, we used two approaches: the program S-DIVA (Statistical Dispersal-Vicariance Analysis; Yu, Harris & He, 2010), which implements a Bayesian approach to dispersal-vicariance analysis (DIVA; Ronquist, 1997), following the method suggested by Nylander et al. (2008), as well as Lagrange (Likelihood Analysis of Geographic Range Evolution; Ree & Smith, 2008) as implemented in the program RASP v.2.1. The geographic distribution ranges were selected based on present day distributions of the species of Synandreae according to information contained in the World Checklist of Lamiaceae and Verbenaceae (Govaerts et al., 2013). Nine geographical areas were identified following the geographical zones defined by Brummitt et al. (2001), and each included taxon was assigned to one or more of these areas: A: southeastern US except Texas; B: east–central US; C: Texas; D: Mexico; E: southern Canada, North Dakota and Northwest Territories; F: western Canada and north–central US, G: Old World, H: Hawaii, and I: southwestern US. Factors leading to the categorizations of these areas are mostly phytogeograpical or based on existing botanical traditions such as areas covered by well-known Flora projects (e.g., Malesia, East Africa, and Mesoamerica). In some cases, however, political factors have out-weighed botanical considerations. A country (for example, the US), being botanically very diverse to be treated as one unit, has been subdivided along internal political (state or province) boundaries. Both the S-DIVA and Lagrange analyses were performed using the tree file generated from the BEAST analyses of the concatenated dataset comprising of *PHOT1*, *PHOT2*, *COR*, and *PPR* and the default settings, except maximum number of areas was set to 4. We did not select the “allow reconstruction” button, and this allowed the program to calculate the proportions of inferred alternative most-parsimonious ancestral ranges at each node in a tree accounting for topological as well as dispersal-vicariance uncertainties. We mapped the ancestral areas onto the 50% majority rule consensus tree derived from our Bayesian analysis of the concatenated dataset.

To estimate divergence timings, we utilized the program BEAST 1.8.3 (Drummond & Rambaut, 2007) on XSEDE through the Cipres portal (Miller, Pfeiffer & Schwartz, 2010) and the concatenated dataset from the four loci as described above. The settings were the same as described above for our Bayesian analysis. Trace files were loaded into Tracer (Drummond & Rambaut, 2007) to look for an Effective Sampling Size (ESS) greater than 200 for all parameters sampled from the MCMC, and to examine the posterior distributions of all parameters and their associated statistics including 95% highest posterior density (HPD) intervals. TreeAnnotator v.1.8.3 (Drummond & Rambaut, 2007) within the BEAST software was used to summarize the set of post burn-in trees and their parameters (burn-in set to 5,000), to produce a maximum clade credibility (MCC) chronogram showing mean divergence time estimates with 95% HPD intervals. The program FigTree v.1.4.0 (Rambaut, 2008) was used for visualization of the resulting divergence timings. The oldest reliable lamioid fossils so far identified have been described from the Seravallian Age of the Middle Miocene flora of Germany and belongs to *Stachys laticarpa* (seed/fruit) and *Lamium* sp. (13.8–11.6 Million years ago (Mya); Mai, 2001). We used the *Stachys laticarpa* fossil as a calibration point (13.8 Mya) to constrain the crown group of the *Stachys* s.l. clade. To reflect the uncertainty related to the fossil data, we set lognormally distributed priors for our calibration with the values for the offset, standard deviation, and mean set to 13.8, 0.8, and 0.5, respectively.

## Results

**Figure 2:**
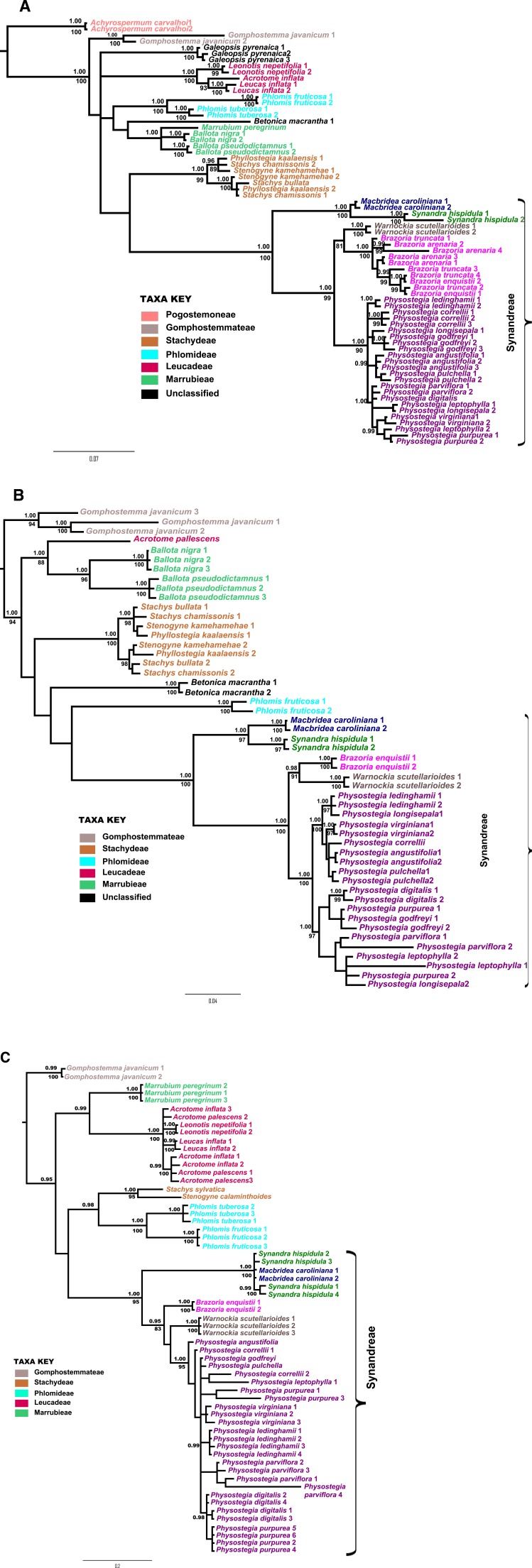
Phylogenetic gene trees. Bayesian 50% majority rule consensus trees obtained from analyses of (A) *PHOT1*, (B) *PHOT2*, and (C) *COR*, respectively. Bayesian posterior probability values ≥0.95 and maximum likelihood bootstrap support values ≥80 are shown above and below the nodes, respectively. Numbers following taxon names refer to different clones from PCR products.

DNA sequence data were collected for a total of 71 samples for the *PHOT1* locus, representing 34 species of Lamioideae, including 17 species of Synandreae. For the *PHOT2* locus, we generated a total of 51 sequences, comprising 25 lamioid species including 14 species representing all of the genera of Synandreae. For the *COR* locus, 64 sequences were included, representing 26 lamioid species and 15 species of Synandreae. Our complete datasets, including gaps, generated from our current study consisted of 564 characters for *PHOT1*, 1,816 characters in the concatenated *PHOT2* dataset, and 352 characters for *COR*, totaling ∼2.7 Kb characters (the raw alignment files in FASTA format for the three loci are provided in Supplemental Information). Including our previously generated *PPR* sequence data (Roy & Lindqvist, 2015) gave a concatenated dataset of ∼3.4 Kb characters. Our results indicated correlation within the overall topologies of the 50% majority rule Bayesian consensus trees and maximum likelihood (ML) trees for the three new datasets (Figs. 2A, 2B and 2C, respectively). Although the sampling for our three new datasets differs due to limitations in the availability of material and success with DNA extraction and amplification, based on the topological congruence in the overall placement of the various species, we expect that the few missing species will group with other members of their respective genera included in the analyses.

**Figure 3:**
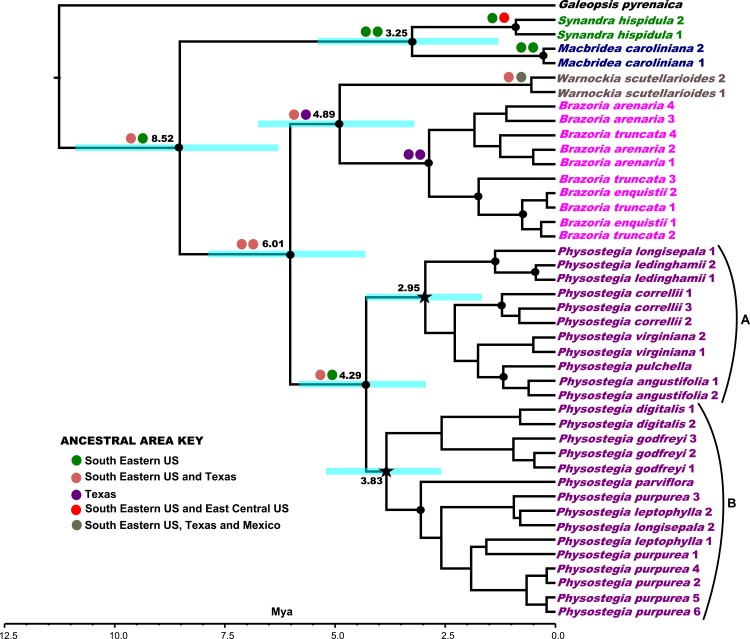
Phylogenetic tree of concatenated nuclear loci. Bayesian 50% majority rule consensus tree obtained from analyses of the concatenated dataset (*COR*, *PHOT1*, *PHOT2*, and *PPR*). Nodes supported by Bayesian posterior probability values (PP) ≥ 0.95 and maximum likelihood bootstrap support (BS) ≥ 80 are labeled with black dots. The black stars represent two nodes (clades A and B) discussed in the text, which both have a PP ≥ 0.90 and a BS > 95. Numbers following taxon names refer to different clones sequenced. The ancestral area reconstructions of Synandreae are labeled as circles next to their respective nodes (see ancestral area key), with left circles representing ancestral area reconstructions from S-DIVA and right circles from Lagrange, respectively. The dates from divergence timing analyses of Synandreae are mapped next to the respective nodes (ages are in Million years). A scale bar has been provided for ages in Million years ago (Mya).

Among the three new datasets, the *PHOT1* phylogeny (Fig. 2A) is based on the most comprehensive sampling of taxa across most of the lamioid tribes. This dataset includes representative taxa from the tribes Pogostemoneae, Gomphostemmateae, Marrubieae, Leucadeae, Phlomideae, Stachydeae, and Synandreae, as well as the unplaced genera *Galeopsis* and *Betonica*. Rooting the tree with *Scutellaria hirta* (not shown), the *PHOT1* phylogeny infers *Achyrospermum radicans* (Pogostemoneae) as sister to all other included Lamioideae (posterior probability value PP = 1.00; bootstrap value BS = 100). *Gomphostemma javanicum* (Gomphostemmateae) is sister to the remaining lamioid tribes, but the latter clade is poorly supported. Although the inter-relationships of the remaining tribes, along with *Galeopsis* and *Betonica*, are unresolved or poorly supported, the tribes themselves, including Synandreae, are strongly supported as monophyletic. In the *PHOT*2 combined phylogeny (Fig. 2B), *Gomphostemma* was used to root the tree in the absence of *Scutellaria* or any representatives of Pogostemoneae. In this tree, *Acrotome* (Leucadeae) and *Ballota* (Marrubieae) form a clade, sister to the rest of the Lamioideae. Within the latter clade, members of Stachydeae are monophyletic (PP = 1.00; BS = 100) and sister to a poorly supported clade comprising *Betonica*, Phlomideae, and a strongly supported Synandreae. In the *COR* tree (Fig. 2C), which used *Scutellaria hirta* to root the tree (not shown), Gomphostemmateae emerges at the base of the lamioid tree, with a clade comprising of Marrubieae and Leucadeae diverging next, and followed by Stachydeae, which forms a well supported clade that is sister to Synandreae, albeit with poor support. The position of Synandreae within Lamioideae remains overall poorly resolved. It is inferred to be sister to Stachydeae based on *PHOT1* (Fig. 2A), sister to *Phlomis fruticosa* based on *PHOT2* (Fig. 2B), sister to a clade composed of Stachydeae and Phlomideae in the *COR* phylogeny (Fig. 2C), and sister to *Galeopsis pyrenaica* within the concatenated tree. Nevertheless, all individual gene trees (Figs. 2A–2C), as well as the phylogeny resulting from the concatenated dataset (Fig. 3), strongly support the monophyly of Synandreae (PP = 0.99 and BS = 100 in *PHOT1*, *PHOT2*, and the concatenated dataset; PP = 1.00 and BS = 95 in the *COR* tree).

The five genera of Synandreae (*Synandra*, *Macbridea*, *Brazoria*, *Warnockia*, and *Physostegia*) are each resolved as monophyletic in all trees. In all phylogenies, *Synandra* and *Macbridea* are resolved as a sister group to the remaining Syandreae members. In phylogenies based on the *PHOT1*, *PHOT2*, and concatenated datasets, *Brazoria* and *Warnockia* are strongly supported as sister groups (concatenated: PP = 0.99; BS = 98), and this clade is in turn sister to *Physostegia* (concatenated: PP = 1.00; BS = 100). In the *COR* tree (Fig. 2C), *Brazoria* is sister to *Warnockia,* which is in turn sister to *Physostegia*, the latter with poor support. This analysis also leaves *Synandra* and *Macbridea* unresolved with respect to each other. In the *PHOT1* and concatenated trees, *Brazoria enquistii* is nested inside *B. truncata* (Figs. 2A and 3). All the individual gene trees, as well as the concatenated dataset, strongly support the monophyly of *Physostegia*; however, relationships among *Physostegia* species are relatively poorly resolved. Hence, in most gene trees there is little support for any paralogy within *Physostegia*, except potentially in the case of *P. leptophylla* (Fig. 2A) and *P. longisepala* (Fig. 2B). This is in contrast to Stachydeae, where the two clones each of *Stachys chamissonis*, *Phyllostegia kaalensis*, and *Stenogyne kamehamehae* in the *PHOT1* gene tree, and also *S. bullata* in the *PHOT2* gene tree, are resolved into two separate clades, pointing to the presence of possible paralogy. In the phylogeny from the concatenated dataset (Fig. 3), all species of *Physostegia* are resolved into two main clades (clades A and B; Fig. 3). Clade A (PP = 0.96, BS = 98) comprises *P. longisepala* (clone1), *P. ledinghamii*, *P. correlli, P. virginiana*, *P. pulchella*, and *P. angustifolia*, whereas clade B (PP = 0.93, BS = 100) comprises *P. godfreyi*, *P. digitalis*, *P. parviflora*, *P. leptophylla* (both clones), *P. longisepala* (clone 2), and *P. purpurea*.

**Figure 4:**
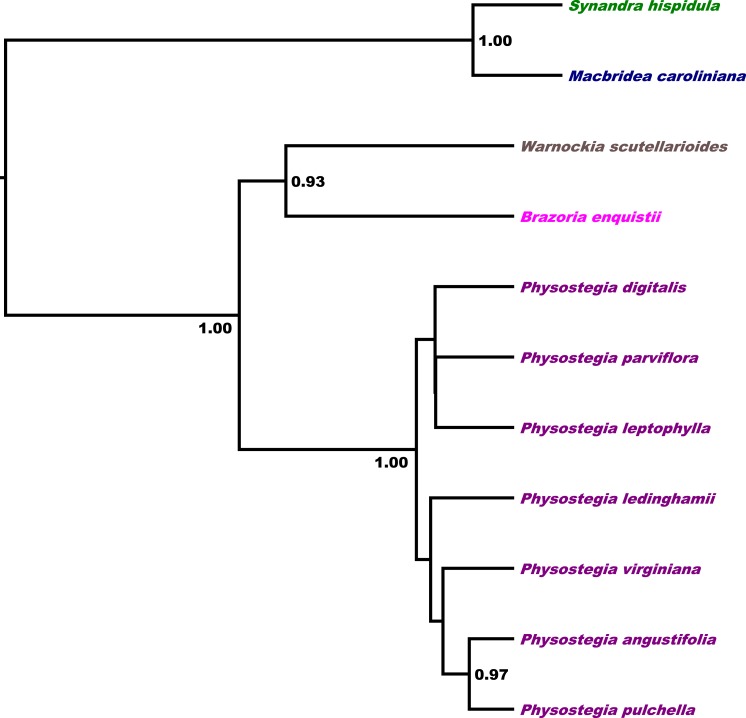
Multi-locus coalescent tree. The coalescence-based tree is inferred from a *BEAST analysis of four nuclear loci (*COR*, *PHOT1*, *PHOT2*, and *PPR*), and a concatenated chloroplast DNA data set (*mat*K, *rp*s16, *trn*L intron, and *trn*L-F spacer). Only nodes with Bayesian posterior probability values ≥0.9 are labeled.

The multispecies coalescence-based tree from the *BEAST analysis of all markers (Fig. 4), corroborates results from previous findings (Scheen et al., 2010; Bendiksby et al., 2011; Roy & Lindqvist, 2015), as well as those from our individual gene trees (Figs. 2A–2C) and concatenated dataset (Fig. 3), supporting *Synandra* as sister to *Macbridea*, which together are sister to the remaining Synandreae (PP = 1.00). *Warnockia* and *Brazoria* form a clade (PP = 0.93), which is sister to a robustly supported *Physostegia* (PP = 1.00).

**Figure 5:**
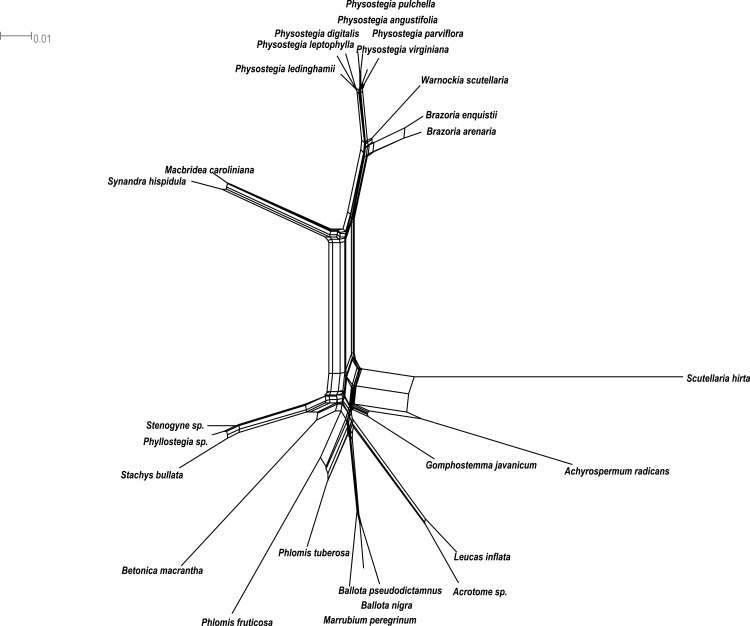
Phylogenetic network. NeighborNet analysis of the concatenated data set for the *PHOT1* and *PPR* loci.

The neighbornet network analysis of the two loci *PHOT1* and *PPR* (Fig. 5) corroborates the clustering of species into their respective tribes and an isolated phylogenetic position of Synandreae separate from the remaining Lamioideae. Within Synandreae, *Synandra* and *Macbridea* are close relatives and separate from its other members of which *Brazoria*, and *Warnockia* are most closely related. No infrageneric phylogenetic structure is resolved among the members of *Physostegia* included here.

Since our current study has limited sampling of lamioid outgroup taxa and tribal interrelationships are incongruent or poorly supported, the results for our ancestral area reconstruction and divergence dating analyses cannot be considered reliable for those taxa. Hence, we have limited our discussion to Synandreae, the focus of the current study. The ancestral area reconstructions (Fig. 3) from S-DIVA infer southeastern US and Texas to be the ancestral area for the entire Synandreae clade, whereas Lagrange excludes Texas from this area in the origin of Synandreae’s most recent common ancestors. For the *Synandra*-*Macbridea* clade, as well as the *Macbridea* clade alone, both S-DIVA and Lagrange infer southeastern US to be the ancestral area. However, Lagrange also includes east–central US in the ancestral area for *Synandra*, whereas S-DIVA points to only southeastern US to be its area of origin. The ancestral area optimized for the *Warnockia-Brazoria*-*Physostegia* clade in both S-DIVA and Lagrange analyses is southeastern US and Texas. For the *Brazoria*-*Warnockia* clade, the S-DIVA analysis points to southeastern US including Texas as the ancestral area, whereas Lagrange shows a greater probability for Texas alone to be the ancestral area for these two genera. For *Brazoria* alone, both S-DIVA and Lagrange analyses unanimously points to Texas as the ancestral area, whereas for *Warnockia* S-DIVA resolves only southeastern US and Texas to be the ancestral area, while Lagrange points to an ancestral area, which combines southeastern US, Texas, and Mexico. For the *Physostegia* clade, Lagrange points towards southeastern US to be the ancestral area, whereas S-DIVA also includes Texas.

Our time calibrated phylogeny (Fig. 3) infers the entire Synandreae tribe to have started diversifying around 6.3–10.9 Million years ago (Mya), with a mean node age of 8.52 Mya. The genera *Synandra* and *Macbridea* diversified further between 1.3 and 5.4 Mya (mean node age 3.25 Mya). Members of *Physostegia* split from *Brazoria* and *Warnockia* between 4.3 and 7.9 Mya (mean of 6.01 Mya ), and the *Brazoria*-*Warnockia* clade further diversified around 3.2–6.7 Mya (mean of 4.89 Mya). Members of *Physostegia* continued their expansion and diversification around 2.9–5.8 Mya (mean node age of 4.29 Mya).

## Discussion

Phylogenetic relationships among Synandreae and their position within Lamioideae were until recently only investigated with cpDNA and nrDNA markers (Scheen et al., 2008; Scheen et al., 2010; Bendiksby et al., 2011). Our current study reconstructs evolutionary relationships in this group based on multiple low-copy nuclear DNA markers. Although our results corroborate many of the findings from previous research (Scheen et al., 2008; Scheen et al., 2010; Bendiksby et al., 2011), we observe some instances of incongruence. Since low-copy loci are biparentally inherited, there is a possibility that either the paternal or maternal gene copy in hybrid progeny was randomly selected, resulting in conflicting patterns in the placement of some of the taxa in the individual gene trees. Our phylogenetic network from the two loci *PHOT1* and *PPR* also shows signatures of reticulation events throughout the phylogeny, including at the base where the different tribes split (Fig. 5). As has been noted in previous studies, the signatures of ancestral gene flow that may have taken place in deep time could have eroded after a long history of divergence, and a substantially larger amount of data are required to precisely pinpoint those loci, which could have introgressed from one species to another (Leache et al., 2014).

### Monophyly of tribe Synandreae: chromosomal evolution and intergeneric relationships

All gene trees (Figs. 2A–2C), as well as the tree from the concatenated dataset (Fig. 3), unanimously corroborate the monophyly of the New World tribe Synandreae, although its position within Lamioideae, and hence its closest relative, remains enigmatic. This clade of North American (NA) endemics is distinguished from most other lamioid genera by the absence of thick-walled cells in the exocarp (Ryding, 1994). The five member genera—*Synandra*, *Macbridea*, *Brazoria*, *Warnockia*, and *Physostegia*—are also characterized by the presence of villous stamens (Harley et al., 2004) and by the anther thecae either narrowing apically to a sharp point (*Synandra*) or bearing one or more teeth along the suture (the other four genera), though it is not clear whether these two character states are homologous.

Our findings unanimously corroborate the monophyly of *Brazoria* and *Warnockia*, which together are sister to *Physostegia*, a relationship also found by Scheen et al. (2008). *Brazoria* and *Warnockia* were recently recognized as separate genera by Turner (1996), having long been treated as congeneric. *Brazoria*, *Warnockia*, and *Physostegia* share distinctive saclike idioblasts in the leaf mesophyll, a feature not found in *Synandra* and unknown elsewhere in the family (Abu-Asab & Cantino, 1987; Lersten & Curtis, 1998), thus an unambiguous synapomorphy.

The strongly supported sister-relationship between *Synandra* and *Macbridea*, which form a clade that is sister to the rest of Synandreae, was also encountered in a nuclear phylogeny based on the *PPR* locus alone (Roy & Lindqvist, 2015), but not in studies based on cpDNA and nrDNA regions (Scheen et al., 2008; Scheen et al., 2010; Bendiksby et al., 2011). In these latter studies, *Synandra* emerged as sister to the rest of Synandreae. There is non-molecular support for both phylogenetic hypotheses. Previous chromosomal studies (Cantino, 1985a) demonstrated that *Macbridea* and *Synandra* have the same chromosome number (2*n* = 18). They also share a derived androecial character—the outer thecae of the anterior stamens are fused (for pictures of this feature in *Synandra*, see Cantino (1985b)). Chromosome numbers based on *x* = 9 are uncommon in subfamily Lamioideae and may be a synapomorphy for a clade comprising *Synandra* and *Macbridea* (Cantino, 1985a). However, in leaf shape, texture, and indumentum, *Macbridea* is much more similar to *Brazoria*, *Warnockia*, and *Physostegia* than to *Synandra* (Cantino, 1982). The leaves in the former four genera are usually lanceolate to elliptical or oblanceolate (rarely ovate and never cordate), narrowing to a cuneate to rounded base, have a firm, semi-succulent texture, are glabrous or at most sparsely puberulent, and at least the upper leaves are sessile. In contrast, the leaves in *Synandra* are broadly ovate-cordate, membranaceous, villous, and petiolate below the inflorescence. Furthermore, Cantino (1990) suggested that absence of anomocytic stomata is a synapomorphy of a clade comprising *Macbridea*, *Brazoria* (including *Warnockia*), and *Physostegia*. It is thus evident that *Macbridea* shares conflicting sets of apparent synapomorphies with *Synandra*, on the one hand, and the *Brazoria*-*Warnockia*-*Physostegia* clade, on the other. A possible explanation for both this character distribution and the inconsistency between cpDNA and low-copy nuclear loci in the placement of *Macbridea* is a scenario involving ancient hybridization between the ancestors of these genera.

*Synandra, Macbridea, Warnockia*, *Brazoria*, and *Physostegia* are characterized by base chromosome numbers *x* = 9 (2*n* = 18), *x* = 9 (2*n* = 18), *x* = 10 (2*n* = 20), *x* = 14 (2*n* = 28), and *x* = 19 (2*n* = 38, 76), respectively (Cantino, 1985a). Although it has been suggested (Gill, 1981) that the ancestral chromosome number in Lamiaceae is *x* = 7, a base number of *x* = 9 in the ancestor of Synandreae could have evolved through aneuploid increase. Similarly, chromosome evolution within Synandreae may have occurred through a series of aneuploidy events (Scheen et al., 2008) from *x* = 9 to *x* = 10, *x* = 14 and *x* = 19 in the ancestors of *Warnockia, Brazoria*, and *Physostegia*, respectively. Increasing chromosome numbers in these genera in comparison to *Synandra* and *Macbridea* has been shown to be positively correlated with a decrease in the chromosome sizes (Cantino, 1985a). Alternatively, the origin of the base chromosome number in *Physostegia* has been posited to be a result of fusion of unreduced gametes (*x* = 9 and *x* = 10) or of polyploidization and merger of normal gametes (Scheen et al., 2008). Hence, the chromosome number of 2*n* = 38 in some *Physostegia* species may indicate tetraploidy, while species like *P. ledinghamii* and *P. leptophylla* may be octoploids (2*n* = 76; Cantino, 1985a). If this hypothesis is correct, *Warnockia* is a good candidate to be one of the progenitors of *Physostegia*, based on its chromosome number (2*n* = 20) and overall morphological similarity. The other progenitor, with 2*n* = 18, is most likely extinct. One can hypothesize that this missing parent of *Physostegia* was the source of its actinomorphic, 5-lobed calyx, a feature not found in any other extant genus of Synandreae. *Macbridea* and *Synandra* would seem to be candidates for the missing parent based solely on their chromosome number. However, there is no morphological evidence for a link between *Synandra* and *Physostegia*. *Macbridea* and *Physostegia* do share a few character states that are not found in *Warnockia*: a rhizomatous perennial habit, mid-stem leaves lacking capitate-glandular hairs, and filaments roughly equal in length (Turner, 1996), suggesting that *Macbridea* might be the other progenitor of *Physostegia*. However, all three of these character states are so widespread in Lamioideae that they could easily be plesiomorphic in Synandreae and thus do not provide convincing evidence for a special relationship between *Macbridea* and *Physostegia*.

### Infrageneric relationships in Synandreae

Both *Synandra* and *Warnockia* are monotypic genera and only one of the two species of *Macbridea* was included in this study. Hence, in our study, only *Brazoria* and *Physostegia* include multiple infrageneric species. Although the resolution within *Brazoria* is not well supported there is some indication that *B. enquistii* may be nested inside *B. truncata*, and hence support for combining these two species. *Brazoria enquistii*, which was recently described (Turner, 2003), is morphologically similar to *B. truncata*, from which it differs in having longer floral bracts with more pronounced ciliation, shorter internodes, and distinctions in the lobes of the calyx.

Our phylogeny of the concatenated dataset assembles all *Physostegia* species into two clades (labeled A and B in Fig. 3). Although we are aware of no morphological synapomorphies for either of these clades, previous morphological studies (Cantino, 1982) have suggested interspecific relationships that receive support in some of our analyses. For example, a close relationship between *P. pulchella* and *P. angustifolia* is indicated (within clade A in Fig. 3 and strongly supported in Fig. 4), corroborating Cantino’s (1982) morphology-based studies. One of the two octoploid species, *P. leptophylla*, which was speculated to be a polyploid derivative of a hybrid between *P. purpurea* and *P. viriginiana* in previous studies (Cantino, 1982; Scheen et al., 2008), groups with both of these species in one of our analyses (Fig. 2A) and with *P. purpurea* in others (Figs. 2B, 2C and 3). However, our results provide only modest support for this hypothesis because *P. leptophylla* also groups with *P. longisepala* in three analyses (Figs. 2A, 2B and 3) and with *P. digitalis* and *P. parviflora* in the multi-locus coalescence-based analysis (Fig. 4). Cantino (1981) and Scheen et al. (2008) also hypothesized a hybrid origin for the other octoploid species, *P. ledinghamii*, involving *P. virginiana* and *P. parviflora* as parents. Although *P. ledinghamii* and *P. virginiana* group within the same clade (A) in the concatenated phylogeny (Fig. 3), our study does not support a close relationship among these three species. On the other hand, a close relationship is suggested between *P. ledinghamii* and *P. longisepala* (Figs. 2B and 3), a relationship also shown in Scheen et al.’s (2008) study, where these two species grouped closely in the 5S-NTS tree. This relationship, however, is not supported by cpDNA, morphology, or geographic distribution. It is also worth noting that a second *P. longisepala* clone groups with *P. leptophylla* (clade B in Fig. 3). It is possible that the different phylogenetic positions of these two *P. longisepala* clones reflect paternal ancestries of the involved species, but further studies with greater number of clones and individuals are needed to support such a hypothesis.

### Biogeography of Synandreae: migration and diversification

*Synandra* and *Macbridea*, which together form a sister clade to the other three genera of Synandreae, are largely confined to southeastern USA, but the range of *Synandra* also extends north into northern West Virginia and central Ohio and Indiana (Cantino, 1985b). *Brazoria* and *Warnockia* are found in south–central US, with *Brazoria* endemic to the eastern half of Texas and *Warnockia* occurring mostly in central Texas with a few outliers in eastern Texas, southern Oklahoma and Coahuila in northern Mexico (Turner, 1996). The most widespread genus, *Physostegia* with 12 species (Cantino, 1982), is extensively distributed across North America, stretching from northern Canada to northern Mexico. However, seven of the 12 species occur in a region comprising southeastern Texas and southwestern Louisiana, suggesting that this area is the center of diversity for this genus (Cantino, 1982). Our ancestral area reconstruction (Fig. 3) supports a scenario in which southeastern US, either including or excluding Texas, is the area where the most recent common ancestor (MRCA) of Synandreae most likely evolved. From this original center of diversity, migration and diversification took place northward and westward.

Roy & Lindqvist (2015) investigated the biogeography of the tribes of Lamioideae, and this fossil-based molecular dating suggested that the MRCA of Synandreae diversified in the New World (NW) from Old World (OW) relatives sometime during the Mid-Miocene epoch. Our current study points to a comparable, although slightly younger, origin around the Late-Miocene/Tortonian age. Since Synandreae appear to be phylogenetically isolated from other lamioid groups, and no well-supported extant sister group of Synandreae has been determined (Scheen et al., 2008; Scheen et al., 2010; Bendiksby et al., 2011; Roy & Lindqvist, 2015), it is likely that several lineages, phylogenetically intercalated between Synandreae and the other extant Lamioideae, have undergone extinction. The Miocene epoch was characterized by extreme climatic optima, with major long-term cooling strongly affecting the distribution and establishment of modern terrestrial biomes (Kürschner, Kvaček & Dilcher, 2008). Atmospheric carbon dioxide variations during the Miocene led to changes in the structure and productivity of terrestrial biomes by affecting their photosynthesis (Flower & Kennett, 1994). East Antarctic ice sheet growth and polar cooling also had large effects on global carbon cycling and on the terrestrial biosphere, including aridification of mid-latitude continental regions (Kürschner, Kvaček & Dilcher, 2008). Such cool-dry cycles of the Miocene epoch could have caused the extinction of some of the closest OW relatives of Synandreae. Numerous biogeographic studies have emphasized the origins and diversification patterns of widely disjunct plant groups in the Northern Hemisphere (NH) (Tiffney & Manchester, 2001; Wen, 1999; Wen, 2001; Donoghue, Bell & Li, 2001; Donoghue & Smith, 2004), and three different biogeographic patterns have been hypothesized for their current distributions. The first pattern suggests that there was an extinction of a once-continuous Arcto-Tertiary, Tethyan or boreal flora, giving rise to the current disjunct distributions of some genera (Mao et al., 2012; Sun, Mclewin & Fay, 2001). The second pattern posits that a majority of genera showing disjunct distributions had their origin on the Qinghai Tibetan Plateau (QTP) and adjacent regions in Asia, later migrating to and colonizing other NH regions (including the Arctic), where they gave rise to derivative species (Xu et al., 2010; Zhang & Fritsch, 2010; Zhang, Kang & Yang, 2009). The third pattern assumes the origin of the groups in other regions of the world with subsequent diversifications on the QTP after the arrival of their ancestors there (Liu et al., 2002; Tu et al., 2010). The absence of a clear extant sister group of Synandreae, presumably due to extinction, is most consistent with the first pattern. Regardless, without a better-defined phylogenetic position of Synandreae within extant Lamoideae, inferences about exact biogeographic origins of the tribe are left uncertain.

### Comparison with Stachydeae: exploring causes for the restricted distribution of most members of Synandreae

Stachydeae and Synandreae, the only two lamioid tribes that include NW members, independently colonized the NW via separate migratory events. Members of Stachydeae (belonging to the genus *Stachys*) colonized the NW twice, once during the mid-Miocene and the other during the Pliocene (Roy et al., 2013), whereas Synandreae colonized the NW only once during the Mid-Late Miocene. While the nearest OW ancestors of NW Stachydeae can be confidently inferred, with African and East Asian *Stachys* species grouping closely with the temperate NA and Hawaiian taxa (Lindqvist & Albert, 2002; Roy et al., 2013; Roy et al., 2015), the closest extant OW relatives of Synandreae are still left undetermined. These two tribes contrast sharply in their pattern of diversification within the NW. NW Stachydeae rapidly migrated and radiated in different parts of temperate NA, Mesoamerica, and South America, and they also successfully colonized and diversified in the Hawaiian archipelago, giving rise to one of the largest clades endemic to the islands (Lindqvist & Albert, 2002; Roy et al., 2013; Roy et al., 2015). Members of Synandreae, on the other hand, split into 19 species in five genera, beginning their migration and diversification in the Miocene and continuing further into the Late Pliocene period. Although their diversification timings in North America are parallel to Stachydeae, Synandreae did not spread outside of North America, with most species staying restricted to the southeastern and south–central US, except for one species of *Brazoria* that extends into northern Mexico and one species of *Physostegia* that has reached into northern Canada.

A number of factors, both biological and ecological, could have led to the disparities in the colonization and diversification patterns of the members of these two groups of NW endemics. Polyploidy seems to be one of the leading factors contributing to the widespread distribution of NW Stachydeae (2*n* = 32–84) and the genus *Physostegia* (2*n* = 38, 76) within Synandreae. Numerous studies have been performed on polyploid genome evolution, and these have shown that phenomena such as substantial intra-genomic rearrangement and altered gene regulatory relationships can lead to a certain degree of evolutionary flexibility, allowing for improved success in colonization and establishment in novel habitats (Levin, 2002; Soltis & Soltis, 2000; Wendel, 2000; Wendel & Doyle, 1998; Tate, Soltis & Soltis, 2005). The high-polyploid members of NW Stachydeae and the Hawaiian mints seem to have rapidly radiated and established themselves in novel habitats, carving out new niches, likely as a result of hybridization and polyploidization (Roy et al., 2013; Roy et al., 2015). This includes *Stachys* species derived from both the Miocene and Pliocene colonizations of the NW. We observe similar trends within the genus *Physostegia*, the only polyploid genus of Synandreae, which has been more successful in colonizing a broad geographic range within temperate NA than its diploid relatives, which have remained largely limited to warm-temperate southeastern and south–central NA.

Abiotic factors could also have played an important role in the current restricted distribution of Synandreae. Glacial climates were extremely variable, and it has been postulated that terrestrial organisms respond individually to climate changes (Huntley & Webb III, 1989). A consensus opinion gleaned from palaeoecological studies is that individual species respond to changing environments through their geographical distributions (Webb & Bartlein, 1992). Glacial conditions have helped shape the modern distributions of most plant and animal species (Willis & Whittaker, 2000). Local flora and fauna during glaciations could have survived only within certain protected localities, referred to as “refugia” (Provan & Bennett, 2008). These refugia provided stable microclimates for species to persist. Southeastern US has been highlighted as a refugium for numerous other species (reviewed by Soltis et al., 2006). The geographic distribution of plant species in southeastern USA has been mainly shaped in an east to west pattern by three geographical factors—the Apalachicola River discontinuity, the Tombigbee River discontinuity, and the Appalachian Mountains discontinuity—leading to endemism and climatically determined glacial refugia (Soltis et al., 2006), especially during the Pliocene and Pleistocene. Swenson & Howard (2005) cited instances of contact zones in Alabama, where closely related species or populations emerging from glacial refugia in Florida and eastern Texas/western Louisiana intermingled. However, due to differential tolerance of climatic and edaphic conditions, species emerging from these refugia became fragmented in their distributions, the less tolerant species thriving only within isolated pockets of favorable abiotic conditions. The spread of *Physostegia*, the most widespread genus of Synandreae, may be due in part to its ability to grow in a broad range of edaphic conditions. Cantino (1982) stated that this genus is tolerant of a wide range of soil acidity conditions. As a result of millions of years of weathering and acidification, southeastern NA is largely characterized by acidic, infertile soils leading to relatively small areas of rich, circum-neutral soils (Manthey, Fridley & Peet, 2011). Hence, other genera of Synandreae, which are not as tolerant of acidic soil conditions, may have remained restricted to such pockets of fertile soil, resulting in their current, more limited, ranges. However, further studies are required to document and substantiate this hypothesis, and to investigate other possible causes, such as anthropogenic alterations of habitat conditions, loss of pollinators, and competition with invasive species, that may also have influenced the current restricted distributions of most species of Synandreae.

##  Supplemental Information

10.7717/peerj.2220/supp-1 Supplemental Information 1 DNA sequence alignment of the *PHOT1* locus

10.7717/peerj.2220/supp-2 Supplemental Information 2 DNA sequence alignment of the *PHOT2* locus

10.7717/peerj.2220/supp-3 Supplemental Information 3 DNA sequence alignment of the *COR* locus

10.7717/peerj.2220/supp-4 Supplemental Information 4 Concatenated DNA sequence alignment of four loci: *PHOT1*, *PHOT2*, *COR*, and *PPR*

## Additional Information and Declarations

### DNA Deposition

The following information was supplied regarding the deposition of DNA sequences: GenBank accessions numbers: KT716867–KT717052.

### Data Availability

The following information was supplied regarding data availability: The raw data have been supplied as Supplemental Datasets.
